# Fluorodeoxyglucose (FDG)-Avid Pericardial Effusion With Reactive Mesothelial Proliferation Mimicking Malignancy

**DOI:** 10.7759/cureus.107842

**Published:** 2026-04-27

**Authors:** Mohammadreza Akbarian Khorasgani, Pouriya Katouzi, Melika Khalifeh Hadi, Danial Alekhamis, Emmanuel Agyei, Jingjing Ma, Chuanbao Li, Xiangjuan Liu

**Affiliations:** 1 Department of Emergency Medicine, Qilu Hospital of Shandong University, Jinan, CHN; 2 Department of Medicine, Shandong University Cheeloo College of Medicine, Jinan, CHN; 3 Department of Medicine, Université Constantine 3 Salah Boubnider, Constantine, DZA; 4 Chest Pain Center, Qilu Hospital of Shandong University, Jinan, CHN; 5 State Key Laboratory for Innovation and Transformation of Luobing Theory, Key Laboratory of Cardiovascular Remodeling and Function Research of Ministry of Education (MOE), National Health Commission (NHC), Chinese Academy of Medical Sciences (CAMS) and Shandong Province, Department of Cardiology, Qilu Hospital of Shandong University, Jinan, CHN

**Keywords:** fdg-pet/ct, malignancy mimicker, mesothelial proliferation, multimodality imaging, pericardial effusion, pericarditis

## Abstract

Fluorodeoxyglucose (FDG)-avid pericardial effusion is frequently associated with malignancy, but inflammation and reactive mesothelial proliferation may produce similar imaging and histologic findings, creating diagnostic uncertainty. This overlap may lead to unnecessary invasive procedures if metabolic imaging is overemphasized. A 76-year-old man presented with progressive bilateral lower extremity edema and was found to have a large pericardial effusion. Imaging demonstrated FDG-avid pericardial and pleural thickening with hypermetabolic lymphadenopathy on PET-CT. Serial cytology, bronchoscopic biopsies, and immunohistochemistry did not demonstrate malignancy. Cardiac MRI showed pericardial inflammation without myocardial involvement. The patient was treated with pericardial drainage and corticosteroids, resulting in clinical improvement and resolution of the effusion. This case is unique because FDG-avid pleuropericardial abnormalities, hypermetabolic lymphadenopathy, and reactive mesothelial proliferation closely simulated malignant disease but ultimately represented a non-malignant inflammatory process. FDG uptake in pericardial effusion is not synonymous with malignancy, and PET-CT findings alone are insufficient for diagnosis. Integrating metabolic imaging with pathology and dedicated cardiac imaging can prevent misdiagnosis and avoid unnecessary invasive interventions, particularly in older or frail patients.

## Introduction

Pericardial effusion in older adults spans a broad differential diagnosis, ranging from self-limited inflammatory pericarditis to tuberculosis and malignancy [1]. The likely etiology is often informed by both the tempo of presentation and the character of the pericardial fluid: rapidly accumulating or hemorrhagic effusions more strongly raise concern for malignant, infectious, traumatic, or iatrogenic causes, whereas more indolent nonhemorrhagic effusions often require broader evaluation for inflammatory and other nonmalignant processes [2]. When effusions are large, recurrent, exudative, or accompanied by pleuropericardial thickening and lymphadenopathy, clinicians often escalate evaluation to multimodality imaging and tissue-based testing [1]. F18-fluorodeoxyglucose (FDG) PET-CT can help localize metabolically active pericardial/pleural disease and screen for extracardiac malignancy, but FDG uptake is not specific and may be increased in inflammatory conditions [1,3]. Cardiac MRI adds complementary value by characterizing pericardial inflammation through assessment of thickening and enhancement and by evaluating for concomitant myocardial involvement [1,4]. In parallel, cytology and histopathology remain central for excluding malignant serosal disease; however, reactive mesothelial proliferation can closely mimic mesothelioma, making immunohistochemistry and ancillary markers important for accurate classification [5,6]. This case report highlights a diagnostic pitfall in FDG-avid pericardial disease and illustrates a structured approach integrating metabolic imaging, cardiac MRI, and pathology to avoid misdiagnosis and unnecessary invasive procedures [1-6].

## Case presentation

History of presentation

A 76-year-old man presented with a five-month history of progressive, painless bilateral lower extremity edema that had worsened over the preceding two weeks. He denied fever, chest pain, orthopnea, palpitations, weight loss, cough, or other respiratory complaints. Appetite, sleep, and daily activities were preserved. Three months before the current admission, he had been evaluated at another hospital, where pericardial and pleural effusions were identified, and both were drained, with partial symptomatic improvement. However, bilateral lower extremity edema later recurred and persisted, prompting further outpatient reassessment. Subsequent echocardiographic evaluation at our institution demonstrated a large pericardial effusion, after which he underwent pericardial drainage and was admitted for further diagnostic evaluation and management. On admission, temperature was 36°C, heart rate 65 beats/min, respiratory rate 17 breaths/min, and blood pressure 119/69 mmHg. Cardiopulmonary and abdominal examinations were otherwise unremarkable, with no clinical signs of cardiac tamponade or overt volume overload.

Past medical history

The patient had no known history of hypertension, diabetes, coronary artery disease, tuberculosis, malignancy, or autoimmune conditions. He reported no history of trauma or invasive cardiac procedures before the onset of the current illness, excluding prior recent pericardial and pleural interventions. He had a long-standing history of cigarette smoking, more than 50 years, averaging 10 cigarettes per day. He had a 20-year history of social alcohol use, which ceased several years prior. Family history was noncontributory.

Differential diagnosis

The leading considerations for a large pericardial effusion in a patient with prior pericardial drainage included malignant pericardial disease, such as mesothelioma or metastatic carcinoma, tuberculous pericarditis, chronic bacterial or fungal infection, autoimmune connective tissue disease, and idiopathic or inflammatory pericarditis. The co-occurrence of pleural effusion, PET-avid lymphadenopathy, and mildly elevated cancer antigen 125 (CA-125) raised concern for malignancy. However, negative microbiologic and cytologic studies, the absence of systemic autoimmune features, and histopathologic ambiguity required broader differential consideration. Elevated inflammatory markers supported a possible inflammatory etiology. Reactive mesothelial proliferation, particularly in older adults, was also considered an important histologic mimic of neoplasm.

Investigations

Following outpatient reassessment and confirmation of a large pericardial effusion on echocardiography, the patient underwent ultrasound-guided pericardial drainage and was admitted for further etiologic evaluation. Transthoracic echocardiography demonstrated a large pericardial effusion with preserved left ventricular ejection fraction (LVEF) (55%), mild mitral and tricuspid regurgitation, and no evidence of tamponade physiology. Pericardial drainage yielded serous fluid, with daily drainage volumes decreasing from approximately 100 mL initially to 30 mL before catheter removal. Initial fluid analysis showed monocyte predominance (85%) and absence of red blood cells.

Laboratory results were notable for elevated inflammatory biomarkers: C-reactive protein (63.48 mg/L), erythrocyte sedimentation rate (74 mm/h), and ferritin (413 ng/mL). Troponin I was modestly elevated (138.8 ng/L). Tumor markers, including CA-125 (94.4 U/mL), were nonspecifically elevated. Serum creatinine and liver function were normal, and urinalysis revealed mild hematuria. Hemoglobin and leukocyte counts were within normal ranges.

Extensive infectious disease workup was negative, including smear and culture for acid-fast bacilli, Mycobacterium tuberculosis deoxyribonucleic acid (TB-DNA), T-cell spot test for tuberculosis (T-SPOT) assay, fungal stains, and fungal serologies. No bacterial or fungal organisms were detected in pericardial fluid. Rheumatologic evaluation and autoimmune panel found no supportive features for systemic lupus erythematosus, rheumatoid arthritis, or other connective tissue disorders. Repeated cytologic analysis of pericardial fluid showed abundant lymphocytes and rare atypical mesothelial cells, but no definitive malignant features.

**Figure 1:**
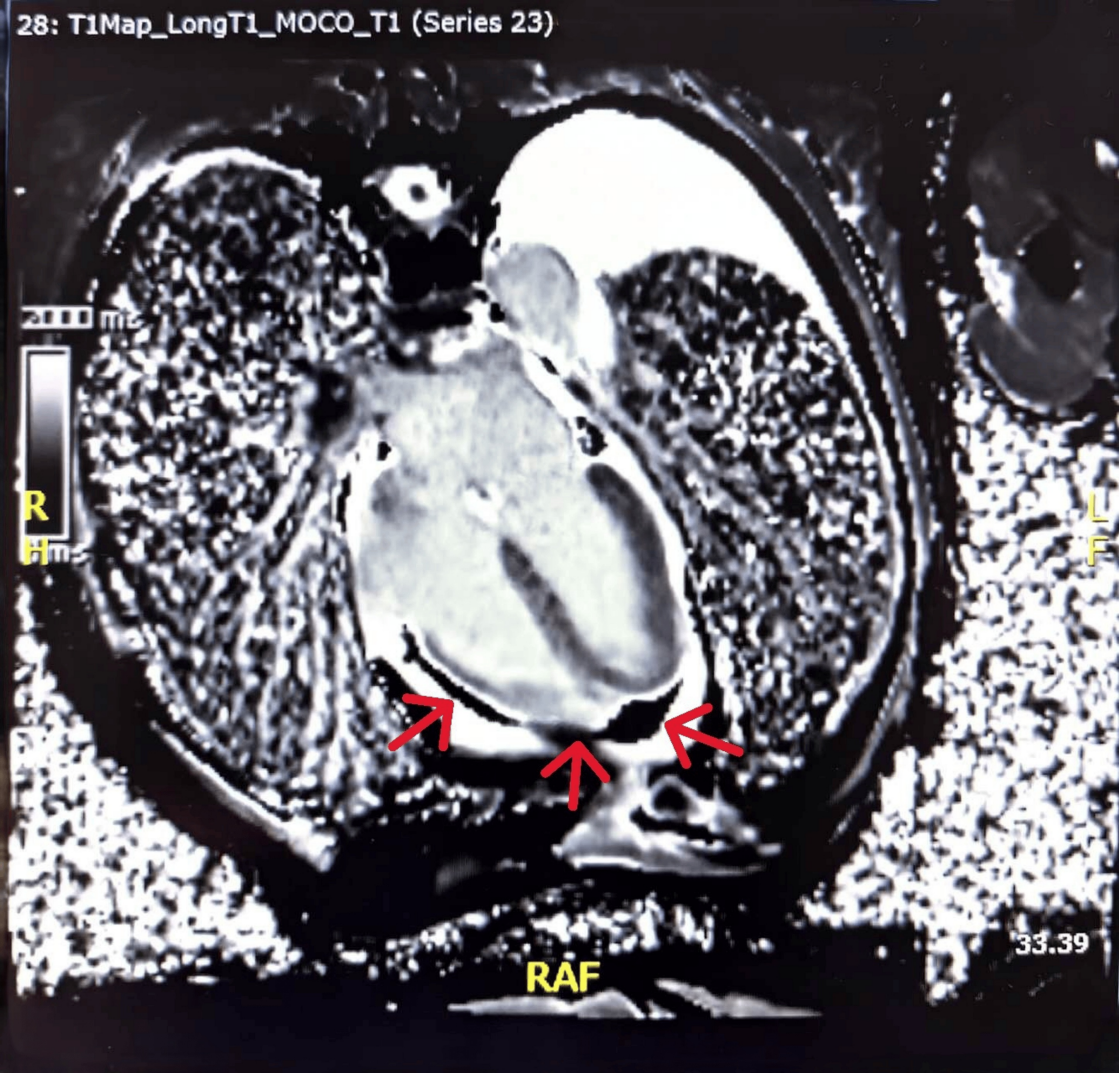
Cardiac MRI Demonstrating Pericardial Effusion and Inflammation Cardiac magnetic resonance imaging (MRI) revealed mild pericardial thickening (up to 5 mm) with late gadolinium enhancement (red arrows), indicating active pericardial inflammation. No myocardial edema or delayed myocardial enhancement was observed, and left ventricular systolic function was preserved (left ventricular ejection fraction (LVEF) 78%). A small left-sided pleural effusion was also noted. These findings supported inflammatory pericarditis without myocardial involvement and helped exclude constrictive physiology.

Because the etiology of the effusion remained unclear after initial fluid analysis, microbiologic testing, cytology, and conventional imaging, further multimodality evaluation was pursued to assess for occult malignancy or metabolically active inflammatory disease. Cardiac MRI demonstrated mild pericardial thickening with delayed gadolinium enhancement and a small, residual pericardial effusion. Myocardial signal and function were preserved, with no late enhancement or edema. These findings were consistent with active pericardial inflammation without myocardial involvement (Figure 1).

**Figure 2:**
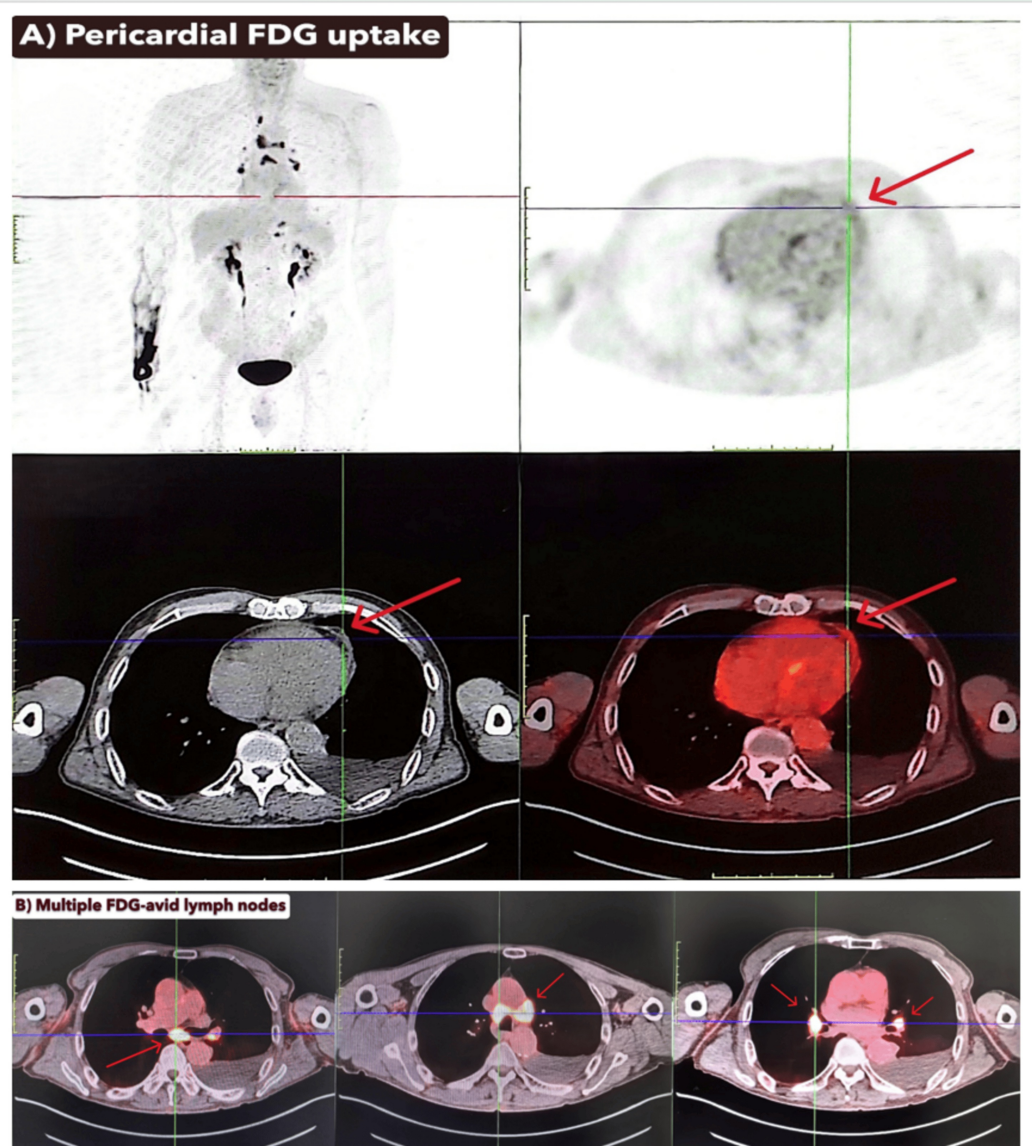
PET-CT Showing FDG-Avid Pericardium and Lymphadenopathy Whole-body F18-fluorodeoxyglucose (FDG) PET-CT demonstrated moderate FDG uptake along the left pleura and pericardium (SUVmax 3.1) (A), with prominent hypermetabolic lymph nodes in cervical, mediastinal, and abdominal stations (SUVmax up to 13.7) (B). No discrete mass lesion or primary malignancy was identified. These findings raised concern for malignancy but were later confirmed to represent a chronic inflammatory response with reactive mesothelial proliferation. SUVmax = maximum standardized uptake value

Whole-body F18-FDG PET-CT revealed metabolic activity in the pericardium and left pleura (SUVmax 3.1), in addition to moderate-to-intense FDG uptake in multiple lymph node stations, including cervical, hilar, mediastinal, and abdominal regions (SUVmax up to 13.7) (Figure 2). Small, benign-appearing pulmonary nodules and bilateral fibrotic changes were also noted. No dominant mass or primary neoplasm was visualized.

Bronchoscopy demonstrated patent airways with pigment deposits but no masses or hemorrhage. Biopsy of bronchial mucosa and mediastinal lymph nodes showed reactive histiocytic proliferation with no evidence of neoplasm. Immunohistochemistry of lymph node samples revealed cluster of differentiation 68 (CD68) positivity with negative cytokeratin (CK), Wilms tumor 1 (WT-1), BRCA1-associated protein 1 (BAP-1), and podoplanin marker (D2-40) staining, excluding metastatic carcinoma and mesothelioma.

Pathologic review of pleural biopsy specimens revealed fibrous tissue with mesothelial hyperplasia and chronic inflammation. Cytology from pericardial effusion was inconclusive, with rare mesothelial cells demonstrating equivocal BAP-1 staining and negative P16, WT-1, and thyroid transcription factor 1 (TTF-1). A secondary review at Qilu Hospital confirmed reactive mesothelial proliferation and excluded mesothelioma.

Management

The patient underwent ultrasound-guided pericardial puncture and catheter drainage over several days with progressive symptomatic relief. Given the inflammatory laboratory profile and absence of infection or malignancy, empiric moxifloxacin was initially started and later discontinued as cultures remained negative. Oral prednisone was initiated at 20 mg daily and gradually tapered. Supportive therapies included low-dose diuretics (furosemide), calcium and vitamin D supplementation, and potassium-magnesium repletion. Close hemodynamic monitoring was maintained throughout hospitalization.

Outcome and follow-up

The patient's blood pressure and heart rate remained stable, and serial physical examinations demonstrated no progression to cardiac tamponade or constriction. Echocardiography on discharge showed a small residual effusion without compromise. The pericardial catheter was removed after confirming minimal output and clinical improvement.

**Figure 3:**
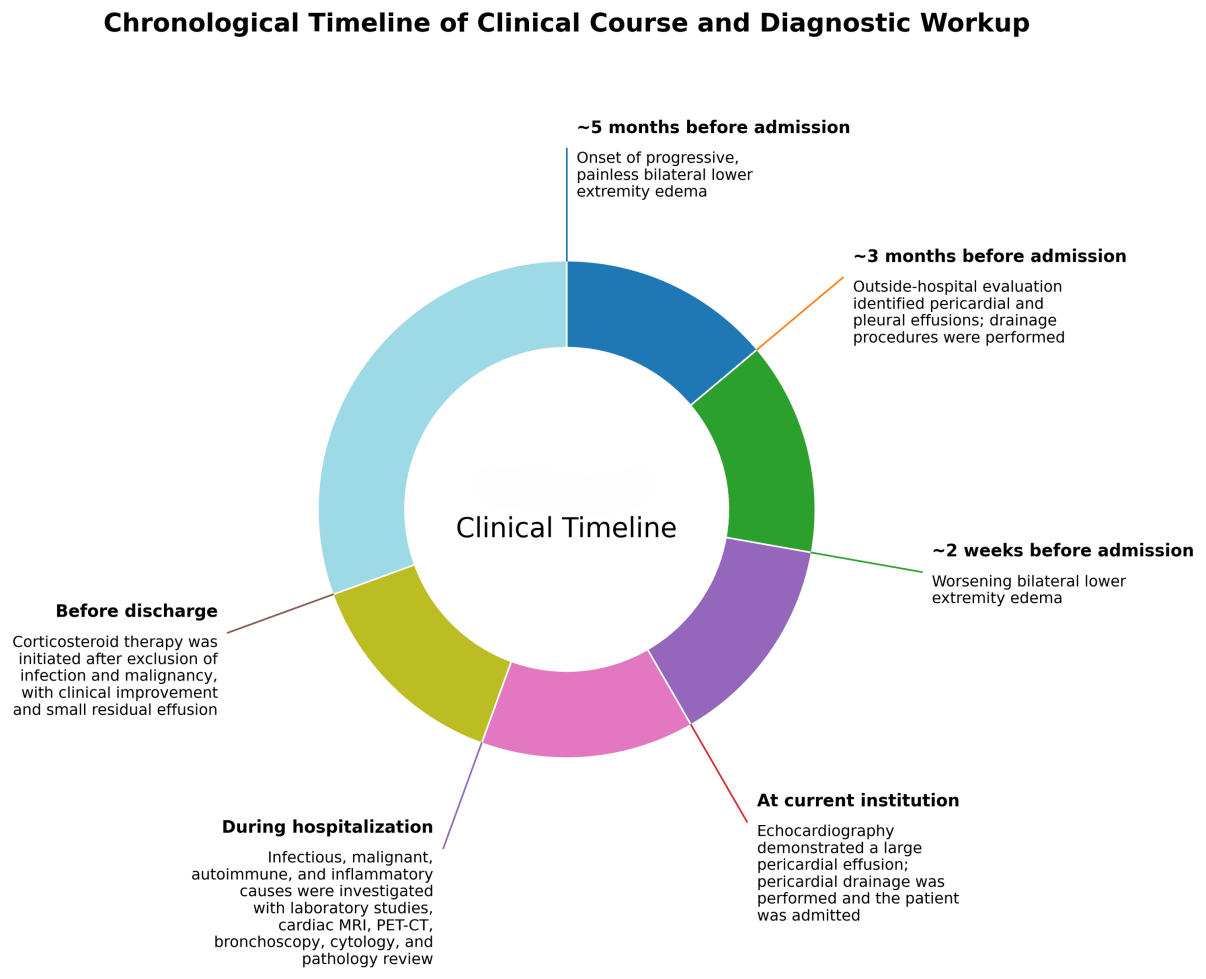
Circular Timeline of Clinical Course and Diagnostic Workup This figure summarizes the chronological sequence of symptom onset, prior outside-hospital drainage procedures, worsening edema before admission, confirmation of large pericardial effusion at the current institution, in-hospital multimodality diagnostic evaluation, and initiation of corticosteroid therapy before discharge with clinical improvement. Image made using Canva (Canva Inc., San Francisco, CA).

He was discharged with tapering corticosteroids, dietary counseling, and instructions for home monitoring of blood pressure and symptoms. Medications included prednisone, calcium carbonate D3, calcitriol, furosemide, and potassium-magnesium aspartate. Scheduled outpatient follow-up at one month was arranged, with instructions to seek care for recurrent dyspnea, chest pain, or edema. At the time of discharge, he was asymptomatic with stable vitals and no signs of volume overload. The chronological sequence of symptom onset, diagnostic evaluation, procedures, and treatment is summarized in Figure 3.

## Discussion

Massive pericardial effusion in elderly patients is diagnostically challenging, particularly when associated with imaging findings that mimic malignancy [7,8]. FDG-PET/CT is often employed to detect neoplastic activity, but FDG uptake is not pathognomonic and may be elevated in a range of inflammatory and infectious conditions [3,9-11].

In this case, PET-avid pericardial and pleural thickening with associated lymphadenopathy and elevated CA-125 initially suggested mesothelioma or disseminated malignancy. However, repeated cytologic analyses failed to identify malignant cells [12,13]. Immunohistochemical profiling was critical in avoiding misdiagnosis [5]. BAP-1 and P16 have emerged as important biomarkers in differentiating mesothelioma from benign mesothelial processes [6,14]. In our patient, retained BAP-1 expression together with negative P16 and WT-1 staining favored a benign reactive process over malignant mesothelial proliferation. Furthermore, chronic pleural and pericardial irritation from previous effusions may have contributed to reactive mesothelial changes.

The diagnostic process also underscores the utility of cardiac MRI in identifying active pericardial inflammation and excluding myocardial involvement [1]. MRI complemented PET-CT findings and helped clarify the nature of disease activity [4].

Management in such ambiguous cases must be individualized [8]. Given the patient's frailty and absence of definitive malignant or infectious pathology, invasive surgical interventions such as pericardiectomy or thoracoscopic biopsy were avoided. Oral corticosteroid therapy offered symptomatic improvement and radiographic regression of effusion. Inflammatory pericarditis with reactive mesothelial proliferation remains an under-recognized but important mimic of malignancy, particularly in older patients with persistent serosal abnormalities.

## Conclusions

FDG-avid pericardial effusion presents a significant diagnostic challenge because inflammatory pericardial disease and reactive mesothelial proliferation can closely mimic malignancy on both imaging and cytology. The uniqueness of this case lies in the combination of FDG-avid pleuropericardial abnormalities, hypermetabolic lymphadenopathy, and reactive mesothelial proliferation that strongly simulated malignant disease but ultimately proved to represent a non-malignant inflammatory process. In this case, PET-CT was not used as an initial or stand-alone investigation, but as an adjunctive modality after initial fluid analysis, microbiologic testing, cytology, and conventional imaging had not established the etiology. This case emphasizes the importance of an integrated diagnostic strategy - combining PET-CT with cardiac MRI, cytopathology, and immunohistochemistry - to avoid overinterpreting metabolic activity as cancer and to prevent unnecessary invasive or oncologic interventions. In clinically stable, older, or frail patients without definitive malignant evidence, a conservative multimodal approach with close follow-up can achieve favorable outcomes.
